# Plant Extract-Synthesized Silver Nanoparticles for Application in Dental Therapy

**DOI:** 10.3390/pharmaceutics14020380

**Published:** 2022-02-08

**Authors:** Omnia Ahmed, Nicole Remaliah Samantha Sibuyi, Adewale Oluwaseun Fadaka, Madimabe Abram Madiehe, Ernest Maboza, Mervin Meyer, Greta Geerts

**Affiliations:** 1 Department of Restorative Dentistry, University of the Western Cape, Bellville 7535, South Africa; 3689306@myuwc.ac.za; 2 Department of Science and Innovation (DSI), Mintek Nanotechnology Innovation Centre (NIC) Biolabels Research Node, Department of Biotechnology, University of the Western Cape, Bellville 7535, South Africa; nsibuyi@uwc.ac.za (N.R.S.S.); afadaka@uwc.ac.za (A.O.F.); amadiehe@uwc.ac.za (M.A.M.); 3 Oral and Dental Research Laboratory, University of the Western Cape, Bellville 7535, South Africa; emaboza@uwc.ac.za

**Keywords:** dental caries, silver nanoparticles, green synthesis, antimicrobial agents, periodontitis, phytochemicals

## Abstract

Oral diseases are the most common non-communicable diseases in the world, with dental caries and periodontitis causing major health and social problems. These diseases can progress to systematic diseases and cause disfigurement when left untreated. However, treatment of oral diseases is among the most expensive treatments and often focus on restoration of form and function. Caries prevention has traditionally relied on oral hygiene and diet control, among other preventive measures. In this paper, these measures are not disqualified but are brought into a new context through the use of nanotechnology-based materials to improve these conventional therapeutic and preventive measures. Among inorganic nanomaterials, silver nanoparticles (AgNPs) have shown promising outcomes in dental therapy, due to their unique physicochemical properties and enhanced anti-bacterial activities. As such, AgNPs may provide newer strategies for treatment and prevention of dental infections. However, numerous concerns around the chemical synthesis of nanomaterials, which are not limited to cost and use of toxic reducing agents, have been raised. This has inspired the green synthesis route, which uses natural products as reducing agents. The biogenic AgNPs were reported to be biocompatible and environmentally friendly when compared to the chemically-synthesized AgNPs. As such, plant-synthesized AgNPs can be used as antimicrobial, antifouling, and remineralizing agents for management and treatment of dental infections and diseases.

## 1. Introduction

Oral diseases such as dental caries (tooth decay) and periodontitis (diseases of the dental supporting tissues) are the most common noncommunicable diseases and affect people of all ages, causing pain, discomfort, tooth loss and disfigurement [1,2]. Dental caries is estimated to affect 60–90% of schoolchildren and nearly 100% of adults worldwide [3]. Dental caries is considered to be the principal cause of tooth loss in children and young adults, and also the primary cause of tooth root breakdown in the elderly [4]. Periodontal disease is classified into four stages, of which severe periodontitis was ranked the sixth most common health condition between 1990 and 2010 [5], and estimated to affect 10% of the global population [6]. Dental caries and periodontitis can progress to systemic infections, leading to infective endocarditis, diabetes, and respiratory infections such as pneumonia [2] if proper prevention and management is not practiced. Both dental caries and periodontitis are polymicrobial in their aetiology and result when the supra- and subgingival microbial communities experience a homeostasis breakdown and undergo dysbiosis, forming a biofilm (plaque) on the teeth and surrounding tissues [7]. Plaque protects the pathogenic microorganisms from the host defence mechanisms, resulting in dental infections [8]. Oral hygiene through the use of antimicrobial and remineralization agents, in addition to mechanical removal of plaque, maybe used to treat and prevent dental diseases [4]. In severe cases, artificial materials are used in restorative procedures (cavity filling, root canal, dental implants, bridges, crowns, dentures) to restore and ensure full function of the oral cavity [8]. However, these strategies are not always successful and restorative procedures could be costly for individuals in low- and middle-income countries. Oral diseases are the fourth most expensive disease to treat, worldwide [3]. In high income countries, the cost of treatment can be covered by patients, with or without the assistance from medical insurance. In contrast, for patients from low- and middle-income countries, treatment may be unaffordable [3]. This, therefore, leaves oral disease prevention and health promotion as the most viable options [9]. The World Health Organization (WHO) Essential Medicines List (EML) programme (2017–2021) recommended that research should focus on developing affordable, safe and environmentally friendly oral hygiene products [10].

In recent years, there is an increasing interest in the use of green synthesized nanomaterials to be included in dental care products. Among the various existing nanomaterials, silver nanoparticles (AgNPs) have gained attention owing to their distinctive physical and bio-chemical properties, as well as their pronounced antimicrobial effects [11]. Moreover, AgNPs due to their larger surface area can be combined with other therapeutic agents to exhibit superior antimicrobial properties in the oral cavity [12]. This review will be devoted to the use of green synthesized AgNPs for the development of novel and improved dental agents to combat oral pathogens, with the goal to arrest and prevent the development of dental caries.

### 1.1. Dental Caries

Dental caries is a chronic oral disease caused by accumulation of microbial biofilm (plaque) on the tooth surface; it occurs as a result of acid build-up that was converted from the free sugars consumed from food products. The acids slowly destroy the tooth enamel and dentine over time, leading to demineralization and subsequently the development of caries. Dental caries has a negative impact on oral-health-related quality of life, and in the advanced stage can lead to tooth loss and systemic infections [6,13]. Among oral diseases, dental caries has a high rate of morbidity across all populations. There is an association between socio-economic and demographic conditions and the incidence of dental caries across all populations [14]. Dental caries not only affects oral health, but can also be associated with systemic and inflammation-related diseases, such as diabetes and respiratory diseases [2,4]; thus, indicating that the prevention and treatment of dental caries are of utmost importance to mitigate this global health problem [4].

#### Pathophysiology of Dental Caries

The oral cavity is a microbiological medium that hosts over 1000 bacterial species [15] that are responsible for metabolic activities in the oral cavity. Primary colonizers are predominantly the anaerobic Gram-positive oral streptococci (e.g., *Streptococcus oralis, Streptococcus sanguinis*, and *Streptococcus mitis*), followed by Gram-positive rods, such as *Actinomyces* species [16]. The oral cavity requires a constant state of equilibrium between the commensal microbial communities to maintain a healthy oral environment [7]. Many factors, which include poor oral hygiene, change in the microflora (cariogenic bacteria), low salivary flow, insufficient exposure to fluoride, diet (high sugar consumption), and immunodeficiency, can alter the oral microbiome homeostasis, and consequently lead to the development of infections [13,14]. The shedding of the oral epithelial lining occurs three times a day, which is an effective method to reduce bacterial adhesion, while the non-shedding surfaces, such as teeth, dentures, and dental implants, are prone to biofilm formation [17].

Dental caries progress when acidogenic bacteria and fermentable carbohydrates interact with host factors such as teeth and saliva. The bacteria adhere to the dental surfaces via adhesion receptors and charge interactions among other mechanisms [16]. The initial attachment of bacteria to dental surfaces is preceded by the formation of the pellicle which consists of glycoproteins (mucins), phosphoproteins, histidine-rich proteins, proline-rich proteins, α-amylase, bacterial glucosyltransferase, etc. [18]. The formation and composition of the pellicle vary from one surface to another [19]. Imbalance in these factors causes fluctuations in plaque pH as a result of increased bacterial acid production and decreased buffering action from saliva and the surrounding tooth structure. Thus, there is a dynamic balance between the tooth surface and its surrounding environment. If this balance is disturbed, the pH drops below a critical value (<6.5–5.5) and the demineralization of tooth structures (enamel, dentine, or cementum) occurs [20]. Demineralization results in dissolution of the major component of tooth enamel and dentine (hydroxyapatite) leading to enamel softening. This appears as a chalky white spot lesion (WSL) on the tooth. At this point, the enamel surface is sound, and the lesion is reversible. If the minerals continue to be lost because of increased acid production, the lesion changes into a black discoloration and subsequent cavitation. If the lesion progresses, large areas of the tooth can be lost [20]. Demineralization is preceded by remineralization, a natural repair process in which minerals such as calcium, phosphate and fluoride from saliva are deposited into the damaged area [21]. However, the natural remineralization process is not always as successful, especially for the larger lesions [22]. Moreover, fluoride-mediated salivary remineralization is limited to the outer tooth structure [23], and remineralization is typically up to 10 times slower than the demineralization process. It is estimated that about 5 h of remineralization are needed to repair a 0.5 h demineralization episode [24]. Additional extrinsic sources of calcium and phosphate ions are then required to accelerate the natural remineralization process. Both demineralization and remineralization processes happen concurrently during the day, with the long-term disequilibrium between the two processes resulting in the development of dental caries [25].

### 1.2. Prevention of Dental Caries

Strategies intended to prevent and arrest the formation and avoid recurrence of carious lesions and cavities include good oral hygiene, diet control, use of saliva substitutes, fluoride, and non-fluoridated and antimicrobial agents [26,27]. These preventive strategies are summarized in Table 1. Proper oral hygiene by itself can neither fully protect against caries or eliminate the biofilm, because biofilm formation starts developing immediately after tooth brushing. It might take time for the microorganisms that are responsible for demineralization to be removed by brushing alone [28]. Regular mechanical plaque control through proper tooth brushing twice daily, can help maintain the composition of the oral microbiome in a healthy state [26], remove plaque in proximal caries [27], and reduce caries. However, mechanical plaque control measures without fluoride are not successful for the prevention of dental caries [29]. Hence, the inclusion of fluoride or agents that delay oral biofilm formation or its symbiosis in the toothpaste may be useful [30]. Fluoride prevents damage to the tooth structure by inhibiting dental caries through remineralization of early lesions [24]. It exerts its antimicrobial effect by forming hydrogen fluoride that diffuse into the bacterium and inhibits its metabolism [28]. When used at the recommended levels—0.7 mg for toddlers, 3 mg for adult women and 4 mg for adult men [31]—fluoride is safe and effective for the prevention of dental caries [27]. Furthermore, the WHO recommended that fluoride toothpaste should be included on the core list of the EML, with fluoride concentration of 1000–1500 ppm, because of its proven activity in this range [32].

Non-fluoridated agents, amorphous calcium phosphate (ACP) and casein phosphopeptide-ACP (CCP-ACP), have been proposed as alternatives to fluoride for the prevention of caries. ACP accumulates on the tooth surface, which then buffers the free calcium and phosphate ion activities, preventing demineralization and enhancing remineralization [48]. CPP have antibacterial and buffering effects on plaque by inhibiting the growth and adherence of *S. mutans* and *S. sorbinus* [27,49]. CPP-ACP products have improved remineralization and anticaries effects when compared to fluoride-containing products [50,51,52,53,54]. However, recent clinical trials revealed that some of these agents should be used in conjunction with fluoride rather than as an alternative [27].

Dietary modification by reducing sugar content to ≤ 10% of total energy intake [9] can dramatically reduce caries incidence [32,55]. Dietary restrictions require a lifelong commitment and are seldom adhered to by patients [24,56]. Insufficient production of saliva as a result of aging, systemic medical conditions, or chemo/radiotherapy lead to delay in the removal of sugary or acidic foods, and consequently biofilm dysbiosis and overgrowth of *Candida* species [57]. In cases of dry mouths, saliva substitutes are used to stimulate saliva secretions to clean the oral cavity and increase the pH buffering capacity [35].

Antimicrobial agents, such as chlorhexidine (CHX), sweeteners, antimicrobial peptides (AMPs), pre- and probiotics, prevent dental caries by altering the dental plaque to be less cariogenic. CHX prevents dental caries by suppression of salivary *S. mutans* [26]. A major reduction in caries in high-caries-risk adults was observed by using 0.12% CHX mouth rinse in conjunction with daily fluoride toothpaste [27]. Sweeteners such as Stevia and Xylitol have anticariogenic properties. Stevia is active against *S. mutans*, *S. sorbinus*, *L. acidophilus*, and *C. albicans*. The indications for using Stevia in the prevention of caries is still under consideration [49]. Xylitol is a natural anti-cariogenic derived from plants and agricultural materials. It acts by disrupting the energy production processes of *S. mutans*, leading to cell death [43]. AMPs became popular due to their selective activity against *S. mutans* and maintaining a balanced oral microbiota for long-term caries protection. Their exact mechanism is not well understood, but membrane disruption and subsequent interference with intracellular targets are thought to be the main processes [58]. AMPs can reduce *S. mutans* count in plaque and saliva without affecting the non-cariogenic oral streptococci [59]. The clinical effect of AMPs is currently limited due to toxicity to normal cells [26].

Pre- and probiotics are used to support microbial diversity in order to obtain a sustained caries-preventive effect. Food products supplemented with live microorganisms, such as *lactobacilli* and *bifidobacteria*, can confer a health benefit to the host [60] and lead to the reduction of *S. mutans* count in saliva [49]. A reduction in initial caries was reported for schoolchildren after drinking milk containing *L. rhamuosus*. Ten randomized placebo-controlled probiotic trials were demonstrated to reverse root caries in preschool children, adolescents, and adults [26]. Probiotic therapy increased diversity of the salivary microbiome after short-term intake of fermented milk containing three probiotic strains. It increased the levels of common commensals, such as *S. oralis* and *S. mitis*, and reduced levels of *S. mutans*, *Fusobacterium* spp. and *Prevotella* spp. So far, there is insufficient evidence for general recommendations of probiotic therapy to modulate the oral microbiome [61]. Prebiotics inhibit the attachment of pathogenic bacteria and stimulate the immune system by altering the pH of the environment [42]. Prebiotics serve as nutrients for beneficial microbes that either inhibit acidogenic and aciduric microbes. The two main sources of alkali in the oral cavity are urea and arginine, which are catabolized by some of the oral bacteria (*S*. *gordonii* and *S*. *sanguinus*) to form ammonia, and thereby elevating the pH of the medium [62]. Arginine in combination with fluoride proved to be effective in arresting dental caries, where it enhanced the arginolytic potential and reduced acidogenicity on early coronal and root caries [63]. Incorporation of 1.5% arginine into toothpaste without fluoride resulted in increased arginine deiminase activity and growth of beneficial bacteria [64]. Numerous clinical studies revealed that CaviStat (arginine bicarbonate and calcium carbonate toothpaste) was able to inhibit caries onset and caries progression in schoolchildren [65]. Subsequently, the Colgate company purchased the right to be the owner of using arginine bicarbonate/calcium carbonate in their toothpaste [24]. The company launched two products containing 1.5% arginine; the Pro-Argin toothpaste against dentin hypersensitivity and anticaries product [24]. All these strategies are extensively reviewed elsewhere [4,66,67].

Despite the significant progress made by preventive strategies to reduce and prevent dental caries, this disease is still considered the most prevalent dental disease in the world [68]. Eliminating carious lesions through restorative procedures in order to improve oral health may not always keep teeth functionality for life. The dental restorations are acceptable from both aesthetic and functional perspectives, although they remain inferior to the natural teeth. Dentures are not stable or comfortable and can affect speech and eating [24]. Preventive or non-operative actions should be used together with the restorative interventions in delivering adequate oral health care [69].

The burden of oral diseases, particularly untreated dental caries, represents a significant public health problem globally. As such, new oral health care products that can produce and ensure sustainable oral health effects are recommended [32]. Currently, there is growing interest in the use of nanomaterial-based systems for dental therapy. In addition to the therapeutic application of nanomaterials, they can also be used as drug carriers for targeted drug delivery, controlled/sustained drug release, improved drug stability and bioavailability [70]. Nanomaterials produced from organic sources are usually preferred over inorganic-based nanomaterials, due to their biocompatibility and biodegradable nature [71]. Some nanomaterials exist as nanoparticles (NPs), which is defined by the International Organization for Standardization as particles with a size range of 1 to 100 nm in one dimension. Organic-based nanomaterials have been used as hydrogels, films, hydrocolloids, and nano-/micro-particles for various biomedical applications [72]. These systems often suffer from low loading capacity and solubility. However, inorganic metallic NPs (MNPs) have an advantage of easily modifiable surfaces, and can easily penetrate the biofilm structure and release their conjugates to destroy the biofilm and inhibit microbial colonization.

### 1.3. Application of Nanomaterials in Dental Therapy

**Figure 1:**
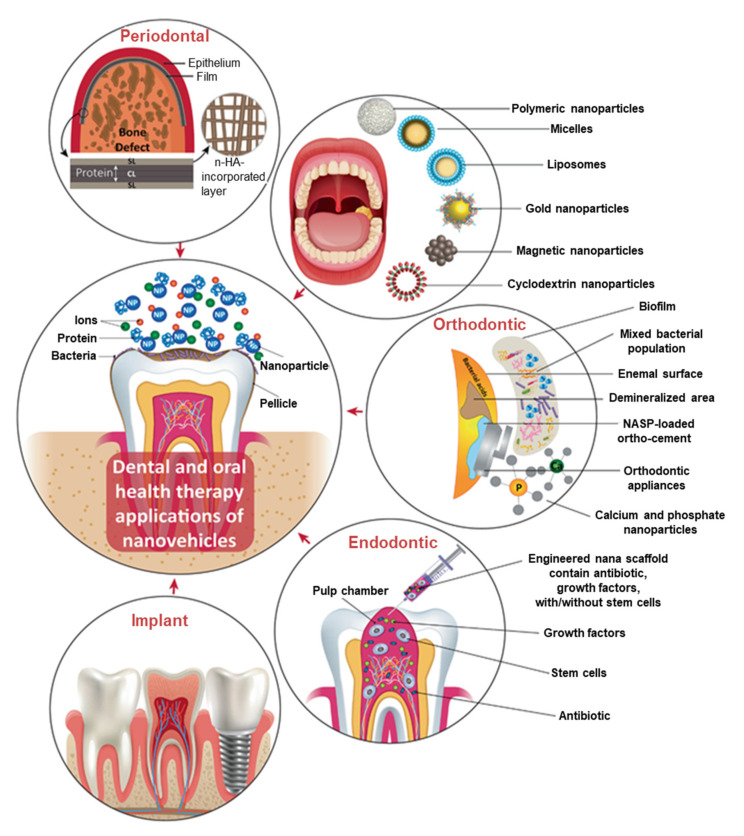
Applications of organic and inorganic nanomaterials in dental care and treatment. Abbreviations: HA: hydroxyapatite, NASP: nano-sized amorphous calcium phosphate. Reprinted with permission from Ref. [76], 2021, Wiley.

Nanotechnology has brought about a lot of exciting and novel applications in various fields, including medicine, through the use of nanomaterials. The use of NPs is now being considered for the treatment and prevention of dental infections and diseases [73]. Various types of NPs exist, and they are broadly classified as inorganic and organic NPs [74]. Inorganic NPs include semi-conductors (ZnO, ZnS, quantum dots), metallic NPs (gold, silver, copper, aluminium, platinum), and magnetic NPs (cobalt, iron, nickel, iron oxide); while organic NPs include carbon (fullerenes, carbon nanotubes) and polymeric (chitosan, liposomes) NPs [74,75]. All these nanomaterials have played a significant role in various experimental dental and oral applications, as shown in Figure 1. These nanomaterials could be incorporated into materials such as, resins, metals, ceramics, etc., used in restorative, prosthetic, endodontics, periodontal treatments, and implantations to prevent and treat oral diseases, including dental caries [76]. In the past few decades, substantial interest and research efforts were directed towards the biomedical applications of MNPs owing to their unique chemical, physical, and biological properties [2,77]. This review will focus on the dental applications of AgNPs synthesized from plant extracts, which are also considered to be green synthesized MNPs.

#### 1.3.1. AgNPs in Dental Therapy

**Figure 2:**
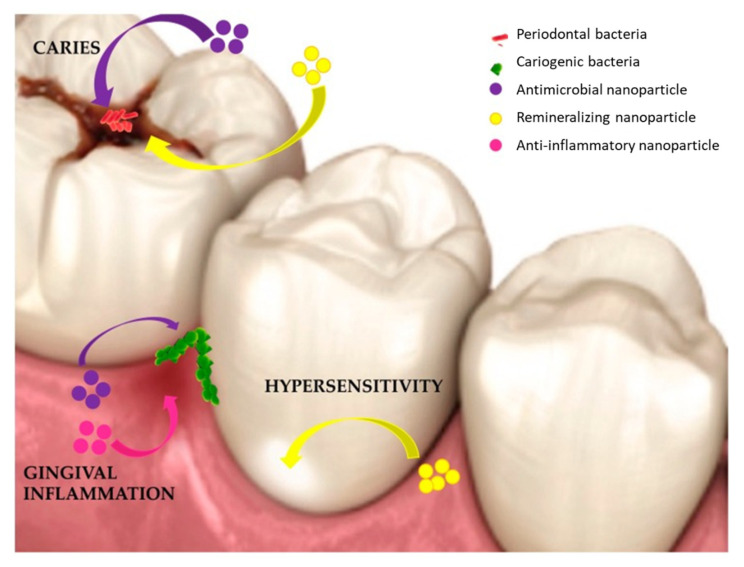
Application of nanoparticles (NPs) contained in oral care products. Reprinted with permission from Ref. [90], 2020, MDPI.

Among the available MNPs, much attention was given towards the exploitation of the biomedically-related effects of AgNPs, particularly its antimicrobial effects. Silver has a long history in dental application, initially in the form of ionic compounds [78], and recently, in NP formulation [79,80,81]. Many studies have demonstrated the antimicrobial effects of 38% silver diamine fluoride (SDF) in the prevention of caries, where the ionic silver and fluoride served as antimicrobial and remineralization agents, respectively [82,83]. However, the high concentration of Ag^+^ that is required to prevent dental caries was found to have an unaesthetic appearance, due to the precipitation of black-coloured Ag on the surface of the tooth. Efforts have been made to overcome this problem in recent years; nanotechnology proved to be effective and showed that by reducing the size of bulk Ag, the surface area was considerably increased. In this way, bioactive molecules can be attached to improve the antimicrobial effects and prevent black staining in teeth [84]. Therefore, there is a growing enthusiasm for use of AgNPs as they provide superior bioactivity [75] due to their distinct physical and biochemical characteristics over Ag^+^ [11,85,86]. Several applications of AgNPs as antimicrobial agents [87] in diagnostics, optoelectronics, and water disinfection agents have been reported [36,88]. The use of AgNPs has been extended to dental applications as antimicrobial [89], anti-inflammatory and remineralization agents [90], as illustrated in Figure 2.

AgNPs have been used in various fields of dentistry (restorative, prosthetic, endodontics, implantology, and periodontology) to prevent caries and treatment of oral cancers. The incorporation of AgNPs into composite resins does not impact on its inherent mechanical and biological properties, but imparts notable antimicrobial properties to the composite material even when low concentrations of the AgNPs are used. It was also demonstrated that AgNPs can reduce the microleakage in the root canal system and can be used as a substitute for sodium hypochlorite (NaOCl) as a canal irrigant. AgNP-based irrigation solution had similar potency to NaOCl in elimination of *E. faecalis*. Although the two solutions were efficient in terms of their antibacterial activity, irrigants with AgNPs showed an added benefit of smear layer removal which may potentiate the ability of AgNPs to interact with the bacteria [91]. The incidence or recurrence of caries due to microleakage can thus be reduced by using composites containing AgNPs with antimicrobial properties and restorative agents [11].

In another study, spherical AgNPs containing polymethyl-methacrylate (PMMA) were able to reduce adherence of *C. albicans* on denture resins [92]. The PMMA-AgNPs were biocompatible and showed no cytotoxic or genotoxic effects on NIH-3T3 mouse fibroblasts and Jurkat cells. The study further indicated that the AgNPs could be added into acrylic resins as an antifungal agent, and possibly reduce the chances of denture stomatitis. Moreover, AgNPs can be favourably used to produce biocidal surface coating onto the titanium implants to prevent periimplantitis, and have also shown potential for osteogenic differentiation [93]. When immobilized on sand-blasted, large grit, and acid-etched titanium, AgNPs did not exhibit apparent cytotoxicity but inhibited the proliferation of *S. aureus* and *F. nucleatum*. These results suggested that by coating titanium implants with AgNPs, the implants can be endowed with balanced antibacterial and osteogenic functions, which bodes well for safe and prolonged clinical applications [94].

Periodontal diseases can also be prevented by AgNPs which can stimulate the regeneration of mammalian cells. The colonization and penetration of guided tissue regeneration (GTR) by *S. mutans, F. nucleatum, Aggregatibacter actinomycetemcomitans,* and *Porphyromonas gingivalis* was studied using membranes impregnated with AgNPs (GTR-AgNPs), which was compared to GTR membrane impregnated with 25% doxycycline hydrochloride (GTR-DOX). The study showed that the bacterial adherence scores and colony forming units (CFUs) for GTR-AgNPs were significantly lower [95]. AgNPs have been shown to possess anticancer activity and prevent WSLs when incorporated into adhesive systems to treat orthodontic patients. The AgNPs prevented crack propagation and improved the fracture toughness within the dental ceramics, which negated the cracking of porcelain restorations with crowns, bridges, and veneers [11].

Chemically synthesized AgNP nanocomposites were also demonstrated to arrest progression of dental caries in 6–10-year-old children in clinical trials [81,82]. A single dose of either AgNP-chitosan-NaF nanocomposite [82] or 5% nano-silver incorporated sodium fluoride (NSSF) dental varnish was administered to school children, and the effects on their teeth were followed for a period of 12 months. The 5% NSSF was composed of polyvinyl pyridoline (PVP) as a dispersant and a commercial sodium fluoride varnish (FLUORITOP™-SR). The clinical cariostatic efficacy of 5% NSSF dental varnish compared to a commercial 38% SDF dental varnish (Saforide^®^, Toyo Seiyaku Kasei Ltd., Osaka, Japan) was evaluated in 6–10-year-old school children presenting carious lesions in primary molars. The active carious lesions were exposed to either 5% NSSF or 38% SDF, at 0, 1, 3, 6 and 12 months. Parameters such as caries activity, depth, size, colour, and presence or absence of pain were noted at the specified time points. Although there was no significant difference between the effects of the two treatments during the 12-month period, 5% NSSF performed similarly and in some cases better than the 38% SDF. In addition, the 5% NSSF did not cause dark staining of the dentinal tissue when compared to the treatment with 38% SDF. Of interest, the safety profile of the 5% NSSF was comparable to the commercial varnish in that no adverse effects were observed in the children subjected to these treatments. This further suggests that AgNP-based oral formulations can be safely used in children. Most importantly, these formulations are cheaper compared to the conventional systems, where 5% NSSF was projected to be eight times less than the cost of SDF [81]. Various AgNP-Fluoride nanocomposites are either recruiting or have completed clinical trials in children or adults, as antimicrobial, demineralization and remineralization agents. A total of 35 studies appear on the Clinicaltrials.gov website under the search for “silver nanoparticles”; some of these studies are summarized in Table 2.

The antimicrobial activities of AgNPs described above were for AgNPs that were synthesised using traditional chemical synthesis, which have shown superior effects against various oral pathogens when compared to other MNPs, such as gold (AuNPs) and zinc oxide (ZnO) NPs. This was demonstrated by Hernández-Sierra et al. who showed that the minimum inhibitory concentration (MIC) and minimum bactericidal concentration (MBC) for these AgNP against *S. mutans* was of 4.86 μg/mL and 6.25 μg/mL, respectively [96]. In comparison, the MIC for AuNPs and ZnO NPs was significantly higher at 197 μg/mL and 500 μg/mL, respectively. AgNP-containing sodium dodecyl sulfate micelle aggregate assemblies were effective against various oral pathogens involved in development and progression of caries (*Pseudomonas aeruginosa*, *Streptococcus gordonii*), root canal infections (*Streptococci* spp.), and periodontal disease (*Enterococcus faecalis*) [86]. Commercially produced AgNPs (Nano-world Company, Calcutta, India) had a MIC between 2.82 and 90 µg/mL against *S. mutans*, *Streptococcus oralis*, *C. albicans*, *L. acidophilus*, and *Lactobacillus fermentum*. The amount of AgNPs required to inhibit bacterial growth was similar to that of conventional antimicrobial agents, such as CHX [97], SDF and 70% isopropanol [86]. Their effect was not diminished when formulated into dental products such as toothpaste. Toothpaste containing AgNP (TruCareNanosilver) had the highest antibacterial efficacy against *S. mutans* compared to those containing chitosan (Conybio Plus Chitosan Dental) and fluoride (Oral B Pro Health). This validates the effectiveness of AgNP-based dental formulations for plaque control and prevention of dental caries or infections [30], and it achieves important clinical effects with a reduced bystander toxicity. Although AgNPs demonstrated many dental health benefits, they can be limited by their instability in various media, and consequently, lose their bioactivities [98]. Their stability in solution can be improved by adding a variety of inorganic or organic [99], synthetic or natural [100], and biotic or abiotic materials as capping agents [101].

#### 1.3.2. Plant-Synthesized AgNPs for Treatment of Dental Diseases

Several methodologies have been employed for the synthesis of MNPs with different sizes and shapes. Generally, MNP synthesis is achieved by various physical and chemical methods, such as laser ablation, lithography, chemical vapor deposition, sol-gel technique and electro-deposition. However, all these methods are not only costly, but were also reported to produce products that might be harmful to humans and the environment. The methods are discussed in detail elsewhere [74,77,102].

With the challenges and concerns raised about MNPs synthesized using physical and chemical methods, many efforts have been geared towards the development of greener and cheaper methods for the synthesis of MNPs. The biosynthesis of MNPs has been shown to be more cost-effective and environmentally friendly compared to the chemical and physical methods [103], because they use natural products from microorganisms and plant extracts to reduce metal precursors, leading to the formation of MNPs [104]. However, while these are green synthesis methods, MNPs produced by microbiological procedures are time-consuming, and both the synthesis and recovery of the MNPs after synthesis can be costly due to the expenses incurred to maintain the organisms during synthesis. Green synthesis methods which involve first extracting natural compounds from microorganisms or plants and then using these extracts for the in vitro synthesis of the MNPs have also been developed, and those synthesized with plant extracts are rapid, use one step synthesis, and do not require sophisticated purification methods [105]. Plant extract-mediated MNP synthesis is more economical as plant extracts are readily available, and extracts from various plant material, such as leaves, roots, stems, barks, flowers, vegetables, fruits, etc., can be used in the synthesis [77,106,107]. In addition, plant-derived waste such as the peels of fruits can also be used to synthesise MNPs. These plant materials contain various phytochemicals (e.g., alkaloids, terpenoids, phenolic) and secondary metabolites (vitamins, amino acids, enzymes, proteins, polysaccharides, antioxidants) [108] that can act as reducing, capping and stabilizing agents during the synthesis of MNPs, either individually or collectively [109].

#### Synthesis of AgNPs Using Plant Extracts

Plant extract-mediated synthesis of MNPs, including AgNPs, have been widely explored and used for various applications [77]. Parameters such as metal precursor concentration, pH, phytochemical composition, reaction temperature and time largely influence the physicochemical properties of AgNPs, such as size, shape, surface charge and morphology [110,111], but can also influence the bioactivities of the AgNPs. Most studies have reported the synthesis of AgNPs in a basic pH medium which seems to produce NPs with a higher stability [112] and a more monodisperse nature. Other advantages reported for synthesis methods done using a basic pH is the rapid rate of synthesis [113] and enhanced reduction process. It was found that small and uniform-sized NPs were synthesized by increasing the pH of the reaction medium [114,115]. The nearly spherical AgNPs produced with the crude extracts were converted to spherical AgNPs by altering pH. However, synthesis in a very high pH (pH > 11) environment was associated with the formation of unstable AgNPs and NP agglomeration [116]. Additionally, the synthesis of AgNPs using plant extracts can be achieved at higher temperatures, including at boiling point (100 °C), while synthesis using mesophilic microorganism can only be performed at temperatures ≤ 40 °C. Mesophilic microorganism cannot survive at temperatures > 40 °C, and this might affect NP synthesis. At 30–90 °C range, AgNP synthesis occurs at higher rate and produce smaller sized AgNPs [117]. Overall, plant-mediated AgNPs have been synthesized at ambient temperatures (25 °C to 37 °C), which produce NPs that still have bioactivities [110]. Their antibacterial actions are usually attributed to various parameters, not limited to size, surface composition, charge and chemical reactivity [8,73]. The fact that these NPs can be synthesised at lower temperatures means that less energy is required to produce these nanomaterials, which does not only reduce the cost of synthesis, but these methods are also more energy efficient and therefore more environmentally friendly. Plant-extract-mediated synthesis of AgNPs have been achieved with various plants extracts; examples include extracts produced from fruit pulp and peels (pears [107]), vegetables (cauliflower [118]), and some of the plants used in traditional medicine (*Cotyledon orbiculata* [119], *Salvia africana-lutea* [120], *Terminalia Mantaly* [106]).

#### Plant Extract-Synthesized AgNPs for Treatment of Dental Diseases

Green synthesis of AgNPs using plant extracts is one of the rapid, simple, and cost-effective approaches that produced stable and biocompatible NPs. Green synthesized AgNPs can be used as a substitute for chemically-synthesized AgNPs. Several in vitro studies that describe the antimicrobial activity of plant-mediated AgNPs against oral pathogens have been reported. AgNPs synthesized from the leaf extract of *Justicia glauca* showed antimicrobial activity alone and in combination with Azithromycin and Clarithromycin against *S. mutans, S. aureus, L. acidophilus*, *Micrococcus luteus, B. subtilis, E. coli, P. aeruginosa,* and *C. albicans*. These AgNPs were also effective against various microorganisms that are associated with dental caries and periodontal disease, with MIC values between 25–75 μg/mL [104]. In another study, biogenic AgNPs produced from plant extracts of *Azadirachta indica* (*A. indica*), *Ficus bengalensis* (*F. bengalensis*) and *Salvadora persica* (*S. persica*) showed antibacterial activity against *L. acidophilus*, *L. lactis*, and *S. mutans*. The *S. persica* AgNPs followed by *A. indica* were more effective against the oral pathogens than the *F. bengalensis* AgNPs [121]. *Haliclona exigua*-AgNPs showed potential to inhibit biofilms on some of the microbes involved in formation of oral biofilm, i.e., *S. oralis*, *S. salivarius* and *S. mitis* [122].

AgNPs synthesized from the aqueous extracts of three different parts of rice grain— viz., rice bran (RB), rice husk (RH), and rice germ (RG)—showed antimicrobial activity against *S. aureus, E. coli, S. mutans* and *C. albicans* [123]. The AgNPs inhibited the growth of all tested microorganisms [123]. One study showed that the biogenic AgNPs have improved activities compared to the chemically synthesized AgNPs and CHX. The chemical AgNPs were reduced by sodium borohydride (NaBH₄), and two green AgNPs were produced using extracts of *Heterotheca inuloides* (Hi) and *Camellia sinensis* (Cs) [124]. Hi produced smaller and more stable NPs when compared to Cs-AgNPs. The green AgNPs inhibited the growth of *S. mutans* and *L. casei* better than 2% CHX, while the smaller Hi-AgNPs had enhanced antibacterial activity compared to the Cs-AgNPs. [124].

Moreover, AgNPs synthesized from extracts obtained from different parts of the pomegranate fruit alone and with β-calcium glycerophosphate showed the highest antimicrobial and antibiofilm activity against *S. mutans* and *C. albicans* [125]. *Gum acacia*-AgNPs were also shown to have antibacterial action against *E. coli* and *M. Luteus*, while the *G. acacia* extracts showed no inhibition effects [126]. Plant extract-mediated AgNPs not only provide a simple one-pot method for in situ synthesis of AgNPs, but produce highly stable AgNPs as they act as both reducing and stabilizing agents. They can be incorporated in different formulations such as mouth rinse and toothpastes to improve their bio-activities [127]. Moreover, like the chemically synthesised AgNPs, the green AgNPs can be used alone or in combination with other dental agents for sustainable therapeutic effects. Thus, AgNPs produced by a green process may be a promising strategy to produce antimicrobial agents against oral pathogens.

### 1.4. Antimicrobial Mechanism of AgNPs

AgNPs have shown broad spectrum antibacterial effects against a number of microorganisms, including some drug resistant microorganisms. While most of the studies investigating the antibacterial mechanisms of AgNPs has been performed using chemically synthesised AgNPs, it can be expected that green synthesised AgNPs will function in the same way. The bactericidal properties of AgNPs were reported to be size and shape dependent [128], but they are also determined by the dissolution of AgNPs and the release of Ag^+^ ions. The influence of size on the antimicrobial activity of chemically- and biologically-synthesized AgNPs was demonstrated in several studies. The antimicrobial activity of AgNPs was reported to be directly proportional to their concentration and inversely proportional to their size, with smaller AgNPs having a lower MIC. AgNPs in the size range of 1–10 nm were shown to have higher antimicrobial activity, as smaller NPs are able to translocate and penetrate into cells much faster than larger ones. Once inside the bacterial cells, smaller NPs can easily interact with various cellular components resulting in bacterial death [129]. AgNPs with a diameter of 5 and 15 nm presented a four-fold lower MIC against *S. mutans* (MIC = 50 µg/mL) when compared to 55 nm AgNPs (MIC = 200 µg/mL) [130]. An independent study corroborated the efficiency of smaller size AgNPs, where 9.3 and 21.3 nm AgNPs reduced *S*. *mutans* adherence on bovine enamel blocks more effectively than larger AgNPs (93 nm). The effects of the 9.3 and 21.3 nm AgNPs were similar to that of CHX [131]. A reduction of 2.3 log in the number of CFUs of *S. mutans* was also observed in biofilms exposed to 100 ppm of 9.5 nm AgNPs [129].

In addition to size, AgNP activity also depends on their shape [132], suggesting that the surface area and the reactivity of AgNP facets are responsible for their antibacterial activity. Silver nanowires showed the weakest antibacterial activity compared to silver nanocubes and nanospheres, while triangular shaped AgNPs were more effective than the nanospheres and rod-like shapes [133]. The triangular shaped AgNPs had a positive charge, which together with the active facets on a triangular-shaped particle was able to ensure a greater antimicrobial activity [134]. In addition to size and morphology of AgNPs, surface composition also plays a role in the activity of AgNPs. This was demonstrated by AgNPs stabilized with chitosan which had altered antibacterial activity against *S. mutans*. The AgNPs showed activity that was higher than the SDF and similar to CHX [134]. Furthermore, the antibacterial action of AgNPs is associated with its dissolution and release of Ag^+^ ions. There are several physical and chemical conditions that can affect the dissolution process, such as temperature, metal ion concentration, surface composition, pH, medium, ionic strength, availability of the oxygen, shape, and size [99]. Morphological properties of AgNPs are indirect effectors that influence Ag+ ion release [135]. The antibacterial activity of AgNPs in some microbes is also associated with the AgNPs themselves, which have been shown to interact and penetrate the microorganisms more effectively than the release of Ag+ ions [80]. In essence, the antibacterial activity of AgNPs can be regulated by altering or modifying the above properties (i.e., size, shape, and (or) surface composition of NPs), to achieve a desired function [62].

**Figure 3:**
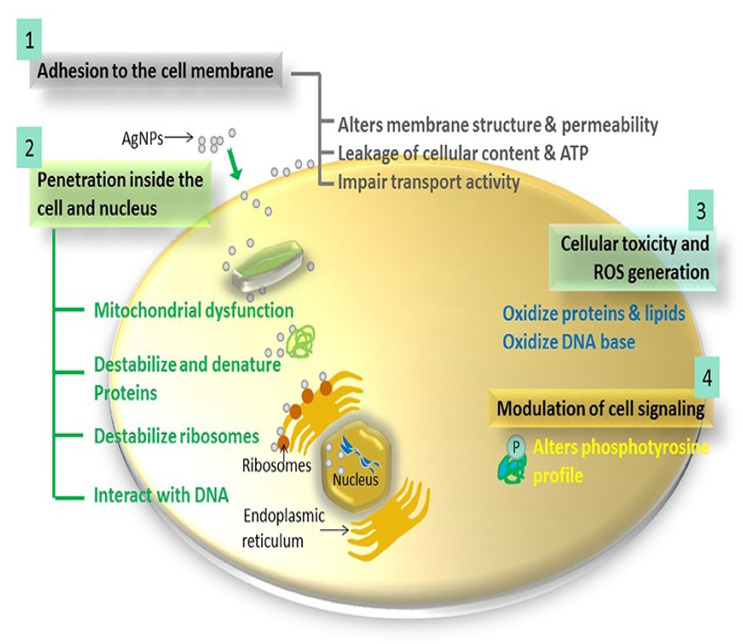
Antibacterial mechanisms of action for AgNPs. Interaction of the AgNPs with the bacterial cell membrane cause damage to the cell membrane and increases membrane permeability (**1**), AgNPs are internalized by the bacteria and interact with cellular organelles and biomolecules (**2**), AgNPs increases ROS production (**3**), and modulate cellular signals leading to cell death (**4**). Reprinted with permission from Ref. [137]. Copyright 2016, Frontiers.

A number of mechanisms pertaining to the antibacterial activities of AgNPs have been proposed, yet the exact modes of action are not thoroughly elucidated [11]. Figure 3 shows four of the common mechanisms described for AgNPs: (1) AgNPs can directly interact with the bacterial cell membrane and alter its permeability, causing cellular damage. They attach to bacterial cell walls by binding to sulfur-, nitrogen-, oxygen- or phosphorus-containing biomolecules, which changes the membrane permeability and consequently causes leakage of cellular contents. (2) When the AgNPs are exposed to the oxygen-rich environment, Ag^+^ ions are released into the cytoplasmic matrix, which then promote microbial death [135]. The Ag^+^ ions induce death by reducing the intracellular adenosine triphosphate (ATP) levels, by inhibition of the mitochondrial activity and protein denaturation [94]. (3) Interaction of the AgNPs with various cellular components induces cellular toxicity through the oxidation of lipids, proteins, and nucleic acids, which will affect the function of these biomolecules and ultimately disturb biochemical pathways. (4) Furthermore, AgNPs were reported to induce dephosphorylation of tyrosine phosphorylated proteins responsible for DNA replication and recombination, especially in Gram-negative bacteria [136]. These combined effects of AgNPs ultimately cause the leakage of cellular components, cell lysis, and death. These mechanisms are extensively reviewed elsewhere [137].

### 1.5. Toxicity of AgNPs

Despite the advantages of AgNPs that recommend them for novel and improved activities in various biomedical applications, their potential toxicity towards humans and the environment is under scrutiny. Metals have been known for centuries to leach into the immediate environment it comes into contact with; this includes the biological environment after ingestion. The daily amount of silver derived from food and water ingested by humans’ range between 0.4–30 µg [138]. With the increasing use of AgNPs in various consumer products, there are also increasing concerns about the possibility that increasing amounts of silver will accumulate in the environment and within humans. AgNPs intended for dental use will be in contact with all structures within the oral cavity, i.e., the teeth, oral cavity tissues, and cells. Thus, these AgNPs might induce serious adverse effects in the surrounding healthy tissues while fighting bacterial infections and oral diseases. There are many reports on the AgNP-induced cytotoxicity in various types of human cells and tissues, including the oral cavity [139,140,141], which makes the safety aspect of AgNPs questionable.

Several factors influence the biological effects of AgNPs; these include size, shape, surface charge and composition. Of clinical importance will be to investigate the relationship between the biological activity, pharmacological activity, and toxicity of AgNPs [142], before it is considered for human application. Studies so far report contradictory results with respect to the toxic effects exhibited by AgNPs within the biological system [143,144]. The toxicity of AgNPs is mainly associated with their dissolution into Ag^+^ ions [145], while their surface composition and charge affect their cellular uptake, translocation, and cytotoxicity. Positively charged AgNPs were more effective against *E. faecalis* and exhibited a high level of cytocompatibility when tested against L929 fibroblast cells [121].

The uptake of AgNPs by cells can induce cellular damage and consequently cell death by damaging vital organelles, such as the nucleus and mitochondria. Interaction of AgNPs with these organelles can cause oxidative stress, DNA damage, chromosomal abnormality, and cell death [146] via necrosis or apoptosis. These phenomena were demonstrated in various human cell lines (keratinocytes, [147], hepatic, neuronal, lung epithelial, and murine stem cells), as well as animal tissues and organs [147]. Repeated exposure to AgNPs can potentiate inflammatory responses, activate the innate immunity [148], and have been associated with renal- [149], hepatic- and genotoxicity [147]. Even at non-cytotoxic doses, repeated doses of AgNPs may be detrimental to vital organs, resulting in their dysfunction and possibly total organ failure [150].

Preclinical studies on rats showed increased accumulation of AgNPs, particularly in the liver, kidney, colon, and jejunum. The accumulation of AgNPs was found to be dose and size dependent [151]. Additionally, oral administration of 56 nm AgNPs in rats resulted in a dose-dependent accumulation of silver in all tissues examined. In this study, a no observed adverse effect level (NOAEL) and lowest observed adverse effect level (LOAEL) were determined at 30 mg/kg and 125 mg/kg, respectively. A higher incidence of bile-duct hyperplasia, with or without necrosis, fibrosis, and/or pigmentation, was observed in AgNP-treated animals. Accumulation of silver in tissues was gender-related, with a two-fold increase in the kidneys of females compared to males [152]. Although AgNPs were present in all the organs examined, higher concentrations were found in the liver and spleen. The high silver concentrations in these organs suggested that the AgNPs were able to penetrate the intestines of the rats. Although the silver content was cleared from most organs eight weeks after treatment, it was still retained in the brain and testicles [153]. Accumulation of the silver in these organs might result in morphological changes that will change the physiological functions of these organs overtime. For example, AgNPs that are taken-up through inhalation may accumulate in the alveolar regions, which might in turn cause lung injury, and the neighbouring organs or those involved in the reticuloendothelial system, such as the liver and kidneys [154,155]. Intranasal administration is convenient for targeted delivery of drugs to the brain, similarly AgNPs administered by this route can be transported to various organs via the neuronal and systemic pathways [154,156]. Although their repercussions are still largely unknown, the long-term retention of AgNPs can exacerbate organ and systemic disorders [154,155].

The antibacterial activity of AgNPs against oral pathogens are significant, but at the same time their potential toxicity cannot be ignored. Studies have shown that the toxicity of AgNPs can be tampered by modifying the surface composition. Surface functionalization of AgNPs improved their biocompatibility compared to the AgNPs without additional capping agents (AgNPs-UC). AgNPs functionalized with lipoic acid (AgNPs-LA), polyethylene glycol (AgNPs-PEG) or tannic acid (AgNPs-TA) induced cell death in a concentration dependent manner. The AgNPs-UC was not cytotoxic to human gingival fibroblast cells at concentrations ≤ 10 μg/mL; after surface modification, the toxicity of AgNPs -LA) and AgNPs-PEG was reduced to 20 μg/mL and 40 μg/mL, respectively. These were the concentrations required for AgNPs-LA to inhibit *Staphylococcus epidermidis and S. mutans* biofilm formation; at the same time, this was not toxic to the human gingival fibroblast cells. Conversely, the other NPs displayed effective antibacterial activities at concentrations that were toxic to the gingival fibroblast cells, i.e., 80 µg/mL for AgNPs-PEG against *S. epidermidis* biofilm formation, and 40 µg/mL for AgNPs-UC against *S. mutans* biofilm formation. The lowest cytotoxicity was observed for the AgNPs capped with LA, the differences in toxicity among the AgNPs clearly demonstrated that the capping agent influenced the AgNP toxicity [142].

In vitro and in vivo toxicity evaluation of 5 nm colloidal AgNPs synthesized with ammonia and polyvinyl pyrrolidone did not induce production of inflammatory mediators (interleukin-1β and -6) at low concentration (≤25 μg/mL), when used in endodontic treatment [157]. When 35 nm AgNPs were used at 50 μg/mL as an irrigant to treat root canal space, they showed antibacterial activity against *E. faecalis*, but no cytotoxicity, whereas concentrations ≥ 80 μg/mL were cytotoxic [158]. Additionally, 10 nm spherical AgNPs were biocompatible in fibroblasts and keratinocytes [159]. The cytotoxicity of AgNPs is influenced by several physicochemical features, including dispersion rate, concentration, surface charge, size, morphology, and composition [160]. The physicochemical properties of nano-silver-based systems raise many toxicological concerns. The experimental results reported to date are insufficient regarding the accurate toxic effects of AgNPs and their related toxicity mechanisms [161]. There are few studies that have examined the cytotoxicity of green synthesized AgNPs to validate their presumed higher biocompatibility. Although studies reporting on AgNPs prepared using green methods are likely biased, these NPs can potentially have similar characteristics to chemically synthesize AgNPs. AgNPs synthesized from *Cotyledon orbiculata* were shown to reduce the viability of THP-1 differentiated macrophages at concentrations between 2.5 and 20 µg/mL [119]. AgNPs synthesized from red pear extracts were not toxic to RAW 264.7 cells at concentrations up to 500 µg/mL, while the AgNPs synthesized from green pear extracts reduced cell viability when concentrations were higher than 125 µg/mL. The fact that these AgNPs showed significant antibacterial effect at concentrations that were not toxic to mammalian cells means that these AgNPs can be considered as biocompatible and probably safe for applications at these doses [107]. Unfortunately, many studies lack appropriate normal cell controls to compare the effects of nanomaterials on normal and diseased cells. Nonetheless, green synthesized AgNPs have shown superior therapeutic activities. *Haliclona exigua*-AgNPs exhibited a dose-dependent cytotoxicity on the human oral cancer (KB) cell line with half the maximal inhibitory concentration (IC50) of 0.6 mg/mL [122]. In addition, AgNPs prepared with *Glycyrrhiza glabra* (*G*. *glabra*) and *Amphipterygium adstringens* extracts inhibited the bacterial growth of *E. faecalis* and the fungus *C. albicans*. Their antiproliferative activity was tested on human epithelial cells, and the results indicated that AgNPs synthesized with *A. adstringens* extract was more toxic to human cells compared to the nanoparticles synthetized with *G*. *glabra* extract [162]. Furthermore, AgNPs synthezied using natural black tea extract had higher cytotoxic activity against ovarian carcinoma when compared to the colorectal carcinoma cell line [154].

The benefits of AgNPs in dental therapy are indisputable; however, there are health and environmental concerns regarding the use of nanomaterials. Therefore, risk assessment measures must be put in place to ascertain and ensure the safety profile of the AgNPs before they are incorporated in consumer products. This will require strategies that will provide localized AgNP effects to reduce bystander cytotoxic effects [163]. Therefore, using natural products in dental formulations as alternatives to fluoride-based containing dentifrices will be more acceptable to consumers as it provides a safety aspect.

## 2. Conclusions

Plant products have been used traditionally for the treatment of many infectious and chronic diseases. The emergence of green nanotechnology has over the years proved that these effects can be enhanced or repurposed when the plant extracts are used as reducing and capping agents of MNPs, including AgNPs. Plant-mediated synthesis of AgNPs is an eco-friendly natural process, which is cost-effective, and renewable for producing innovative, bioactive and biocompatible AgNP-based products for human use. Thus, biogenesis of AgNPs has a potential application in dental therapy; they can be used as antimicrobial agents in various fields of dentistry. They can be incorporated in dental products that are currently used as canal irrigants, toothpastes, mouth wash, varnish, and also in dental restorative implants. Clinical trials of chemically-synthesized AgNPs are ongoing to evaluate their ability to prevent tooth demineralization, dental biofilms and treatment of dental caries. AgNP-based products are already incorporated in various consumer products, and expected to grow exponentially in the coming years. Therefore, it is pivotal that their biodistribution and toxicity is evaluated before their use in human products. Despite these concerns, plant-based AgNPs can be used for development of multifunctional dental care products that can aid in the prevention and treatment of dental infections and diseases.

## Figures and Tables

**Table 1:** Preventive strategies for dental caries.

Caries Prevention Agents	Examples	Formulation Type/Tools	Action	Limitation	Refs
Oral hygiene	Toothbrush Dental floss Mini-brushes Oral irrigators		Delay or prevent oral biofilm formation	Flossing alone may not be effective in reducing caries Personal skills influence the outcome Must be used in combination with toothpaste	[27,29,30]
Diet modification	Sugar reduction	Food	Control biofilm formation by eliminating plaque Lowers the rate of dental caries	Reducing sugar content is less successful Dietary control of sugar is a challenge	[33,34]
Saliva substitutes			Cleans the oral cavity and the pH buffering capacity		[35]
Fluoride		Toothpaste Varnish Gel Mouthwash Tablets Drops Chewing gum Lozenge	Remineralization	Fluoride classified as a neurotoxicantContinuous use can lead to fluoride-resistant strains Excessive use can lead to tooth fluorosis, discoloration, and gastrointestinal problems Skeletal fluorosis can cause disabilities such as chronic joint pain, stiffness of joint, sporadic pain, calcification of ligaments, and osteosclerosis	[24,36,37]
Non-fluoridated (calcium-based) agents	Amorphous Calcium Phosphate (ACP)	Added into a fluoride toothpaste	Remineralization	Reduce root caries Unstabilized ACP promote dental calculus deposition on the teeth ACP interferes with remineralization by removing free fluoride ions in the oral environment	[21,38]
	Casein Phosphopeptide- ACP (CPP-ACP)				
Antimicrobial Therapies	Chlorohexidine (CHX)	Mouthwash	Antimicrobial agent	Brownish staining of the teeth, removable by discontinuing the product Altered taste, persist for several hours after use Oral burning Development of lesions and ulcerations of the gingival mucosa Has a strong, unpleasant taste	[39]
	Probiotics Prebiotics	Probiotics: dairy products, fermented vegetables, sourdough bread, drops, tablets, and lozenges containing various strains	Reduce harmful gastrointestinal microorganisms, discomfort, and stimulate the immune system		[26,40]
		Prebiotics: chewing gum, oral rinse	Stimulate growth and/or activity of bacteria already resident in the host colon Inhibit the attachment of pathogenic bacteria Alters pH of the environment Stimulate the immune system		[41,42]
	Sugar substitute (sweeteners), e.g, Stevia, Xylitol	Chewing gum	Stevia: reduces plaque formation Acts as a healing agent at the periodontium level		[26]
			Xylitol: reduces plaque formation, and bacterial adherenceInhibits enamel demineralization (i.e., reduces acid production)		[43]

Fluoride toothpastes that contain lysozyme, lactoferrin and proteins can modulate oral health by generating hydrogen peroxide and hypothiocyanite, which can reduce pathogenic microorganisms and promote the proportion of bacteria associated with good gum health [44]. However, continuous use of fluoride can drive the development of fluoride resistance in bacteria, such as *S. mutans* [45], *S. salivarius,* and *S. sanguinis* [46], and are not adequate in highly cariogenic oral environments [47]. Moreover, fluoride is classified as an “unapproved new drug” by the US Food and Drug Administration, and as of January 2012, a memorandum to end water fluoridation worldwide was signed [31]. Additionally, fluoride toothpaste, rinses and varnish applications are not universally affordable [31].

**Table 2:** AgNP-based formulations registered for clinical trials.

AgNP Formulation	Study Title	Conditions	AgNP Activity	Status Online	NCT Identifier
AgNPs in vanishing cream	Topical silver nanoparticles for microbial activity	Foot fungal and bacterial infections (tinea pedis, capitis, versicolor)	Antimicrobial	Recruiting	NCT03752424
Innocuous containing gel AgNPs	Topical application of silver nanoparticles and oral pathogens in ill patients	Critical patients in ICU (coma or induced coma)	Antimicrobial	Completed	NCT02761525
AgNPs	Silver nanoparticles in multidrug resistant bacteria	Critically ill patients	Antimicrobial	Completed	NCT04431440
5% NSSF	Nano-silver fluoride to prevent dental biofilms growth	Dental caries	Antimicrobial	Completed	NCT01950546
AgNPs incorporated into the primer orthodontic Transbond XT	Addition of silver nanoparticles to an orthodontic primer in preventing enamel demineralization adjacent brackets	Dental caries	Tooth demineralization	-	NCT02400957
Mouthwash and nose rinse with AgNPs	Evaluation of silver nanoparticles for the prevention of COVID-19	COVID-19	SARS-CoV-2 activity	Completed	NCT04894409
Colloidal silver	Colloidal silver, treatment of COVID-19	Severe Acute Respiratory Syndrome		Recruiting	NCT04978025
Fluor dental varnish with 25% AgNPs	Fluor varnish with silver nanoparticles for dental remineralization in patients with Trisomy 21	Dental caries in Trisomy 21 children	Remineralization	Active, not recruiting	NCT01975545
Nano-silver fluoride solution	Radiographic assessment of glass ionomer restorations with and without prior application of nano- silver fluoride in occlusal carious molars treated with partial caries removal technique	Partial dentin caries removal	Caries arrest prior to glass ionomer restoration	Completed	NCT03193606
Hydrogel/nano silver-based dressing	Evaluation of diabetic foot wound healing using hydrogel/nano-silver-based dressing vs. traditional dressing	Diabetic foot ulcer	Wound healing	Completed	NCT04834245
Central venous catheter impregnated with AgNPs (AgTive^®^)	Comparison of central venous catheters with silver nanoparticles vs. conventional catheters	Central venous catheter-related infections		Completed	NCT00337714
Silver colloid	The effectiveness of topical silver colloid in treating patients with recalcitrant chronic rhinosinusitis	Recalcitrant Chronic rhinosinusitis		Completed	NCT02403479
Nano-silver (SilvaSorb^®^) gel	Efficacy of silver nanoparticle gel vs. a common antibacterial hand gel		Antimicrobial	-	NCT00659204
Nano-silver Fluoride	Remineralization of dentine caries using two remineralizing agents which are nano-silver fluoride and casein phosphopeptides amorphous calcium phosphate	Dental caries	Remineralization	recruiting	NCT04930458
Nano-silver fluoride	Effect of using different varnishes on dentin hypersensitivity; Na fluoride and nano-silver fluoride	Dentin hypersensitivity	Tooth hypersensitivity	Not yet recruiting	NCT04731766
Nano-silver fluoride varnish	P11-4 and nano-silver fluoride varnish in treatment of white spot carious lesions	WSL	Remineralization in permanent teeth	recruiting	NCT04929509
Nano silver fluoride solution	Antibacterial effect of nano silver fluoride vs. chlorhexidine on occlusal carious molars treated with partial caries removal technique	Dental caries	Antibacterial	Completed	NCT03186261
Nano silver fluoride	Clinical and radiographic evaluation of nano silver fluoride vs. calcium hydroxide in indirect pulp treatment of deep carious second primary molars, randomized clinical trial	Deep caries		-	NCT04005872
AgNPs vs. copper NPs	Effect of metallic nanoparticles on nosocomial bacteria	Nosocomial infections	Antibacterial	Recruiting	NCT04775238

## Data Availability

The review article used data from published studies, and the studies are referenced accordingly.
